# Symplasmic Intercellular Communication through Plasmodesmata

**DOI:** 10.3390/plants7010023

**Published:** 2018-03-20

**Authors:** Jae-Yean Kim

**Affiliations:** 1 Division of Applied Life Science (BK21 Plus Program), Plant Molecular Biology and Biotechnology Research Center, Gyeongsang National University, Jinju 660-701, Korea; kimjy@gnu.ac.kr; 2 Division of Life Science (CK1 Program), Gyeongsang National University, Jinju 660-701, Korea

**Keywords:** plsamodesmata, intercellular communication, mobile transcription factor, callose, lipid raft, natural heterografting, receptor-like protein/kinase

## Abstract

Communication between cells is an essential process for developing and maintaining multicellular collaboration during plant development and physiological adaptation in response to environmental stimuli. The intercellular movement of proteins and RNAs in addition to the movement of small nutrients or signaling molecules such as sugars and phytohormones has emerged as a novel mechanism of cell-to-cell signaling in plants. As a strategy for efficient intercellular communication and long-distance molecule movement, plants have evolved plant-specific symplasmic communication networks via plasmodesmata (PDs) and the phloem.

Plasmodesmata (PDs) and the phloem provide short- and long-distance symplasmic pathways to exchange signaling molecules, respectively [1]. PDs are microscopic intercellular organelles carrying multiple membranous nano-channels which symplasmically interconnect neighboring cells and allow movement or trafficking of cellular signaling molecules. PDs provide original intercellular pores that can also be modified and widen to sieve pores found in specialized phloem sieve cells. While intercellular communication is a pivotal process to develop and maintain multicellular systems, the plant cell walls of about 200–1000 nm may function as physical or communication barriers between adjoining cells [2]. In plants, this impediment has been overcome by the evolution of two signaling pathways: apoplasmic signaling mediating interaction between mobile ligands and receptors, and symplasmic signaling involving plasmodesmal molecule movement. In this special issue, Kitagawa and Jackson provide an excellent review on PD-mediated cell-to-cell communication in the shoot apical meristem (SAM), and mechanisms underlying molecular trafficking through PDs [3]. 

## 1. Macromolecular Trafficking through PD

Considering that PD pores have nanometer-scale diameter, it was initially suggested that PDs allow movement of less than about 1 kDa molecules; however, they have shown a great dynamic capacity to allow movement of macromolecular proteins and RNAs [4]. Transcription factors (TFs) are very ideal cell-to-cell messengers that can carry signaling information. KN1-related homeobox (KNOX) TFs such as KNOTTED1 (KN1), SHOOT MERISTEMLESS (STM), and KNOTTED-LIKE FROM ARABIDOPSIS (KNAT1)/BREVIPEDICELLUS (BP) are mobile in the SAM, leaf, and stem. KN1 trafficking is selective, and the homeodomain of KNOX family members was identified as intercellular trafficking motif (ITM) that is necessary and sufficient for its PD-mediated transport [5]. It was suggested that the ability to efficiently traffic for KNOX TFs may have been acquired during the evolution of early nonvascular plants, since the unicellular *Chlamydomonas* homeodomain related to the KNOX TF was unable to move cell-to-cell [6,7].

Their intercellular trafficking is tissue-specific. KN1 protein traffics bi-directionally between L1 and L2 in the SAM, but in the mature leaf, only unidirectional L2-to-L1 movement was detected [8]. Their intercellular movement is required for the normal developmental process; KNAT1 trafficking from the cortex to epidermal cells in stems is required for the determination of plant architecture and epidermal differentiation [9]. The biological significance of TF movement was also demonstrated in WUSCHEL (WUS) case. A WUS-CLAVATA3 (CLV3) negative feedback loop is a key process to maintain the size of a stem cell pool [10]. WUS expressed in the organizing center of the SAM moves to central zone cells and activates CLV3. CLV3 functions as an apoplasmic mobile peptide that is received by a receptor-like kinase/pseudokinase CLAVATA 1 (CLV1) and CLAVATA 2 (CLV2)/CORYNE (CRN) complex to repress WUS expression. Artificial restriction of WUS movement caused *wus* mutant-like phenotypes [11], suggesting that WUS movement is required for the maintenance of the SAM stem cell pool. In addition to mobile TFs, non-TF signaling regulators such as type-A ARABIDOPSIS RESPONSE REGULATOR 7 (ARR7) [12], the CK signaling inhibitor ARABIDOPSIS HISTIDINE PHOSPHOTRANSFER PROTEIN 6 (AHP6) [13], and DWARF 14 (D14), a receptor of strigolactone (SL) hormone in rice [14], were identified as non-cell-autonomous mobile proteins.

Diverse RNAs, such as non-coding small (miRNA and siRNA) and long RNAs, and mRNA, like mobile proteins, also act as mobile signals [15,16]. For example, miRNA394, targeting LEAF CURLING RESPONSIVENESS (LCR), moves from the L1 to the L2/L3 in the SAM [16]. miR394-deficient mutants prematurely terminate the SAM, suggesting the importance of the repression of LCR by miR394 for stem cell maintenance. 

As non-cell-autonomous RNAs, more than 2000 mRNAs were identified from phloem sap using a heterografting system [17,18]. However, the biological function of most mobile RNAs is awaits further characterization. In this issue, Hannapel and Banerjee [19] report that multiple mobile mRNA signals regulate tuber development in potatoes. Several mRNAs, encoding transcription factors from the three-amino-loop-extension (TALE) superfamily, have been found in the phloem and have been shown to move from leaves to roots and stolons to control growth and tuber formation. StBEL5 as well as the sequence-related but antagonistically functioning StBEL11 and StBEL29, function to control growth and tuber development. The phloem transport of all three of these RNAs has been found to be enhanced in short-day conditions. However, the evolutionary advantages of this dynamic and complex signaling pathway, which uses mobile mRNAs that function as both activators and repressors, are still not clear.

## 2. Regulatory Mechanisms of PD Movement

Molecule movement through PDs can be selective (active) or non-selective (passive). Selective protein trafficking requires protein-protein interaction, the involvement of cytoskeletons or partial unfolding of mobile proteins. During PD transport of KN1, STM, and TRANSPARENT TESTA GLABROUS1 (TTG1), Type II chaperonin including CHAPERONIN CONTAINING T-COMPLEX POLYPEPTIDE 1 (CCT) 8, CCT1 and CCT5 was suggested to function [20,21]. An endosome-associated protein, SHORT-ROOT INTERACTING EMBRYONIC LETHAL (SIEL) is required for SHORT ROOT (SHR) movement and interacts with other mobile TFs, CAPRICE (CPC), TARGET OF MONOPTEROS 7 (TMO7), and AGAMOUS-LIKE 21 (AGL21) [22]. Especially, SIEL-dependent SHR movement seems to be assisted by microtubules and endosomes.

In addition to selective trafficking, diffusion-based movement is controlled by PD structure, channel size or frequency, which controls PD permeability [23]. PD permeability can be controlled by callose-dependent or callose-independent modes [3,24]. The former involves the balance of two key enzymes, callose synthases (CalS, GLUCAN SYNTHASE-LIKE (GSLs)) and callose degradation enzymes (beta-1,3-GLUCANASES). The latter includes the Ca^2+^ or reactive oxygen species (ROS) signaling, or involves cytoskeletal proteins. The frequency of secondary PDs, which are formed de novo in post-cytokinetic walls, is reduced by *choline transporter-like 1* (*cher1*) mutation [25] and increased by *increased size exclusion limit of plasmodesmata 1* and *2* (*ise1*, *ise2*) [26]. CHER1 function in the formation of secondary PDs might be implicated by altering membrane composition or the targeting of processing enzymes to PDs [27]. In similar aspects, our review in this special issue focused on the potential role of lipid rafts as regulators of PD callose homeostasis [28]. Lipid rafts are specialized plasma membrane microdomains and are enriched by sterols and sphingolipids. PD containing phospholipid bilayer membranes consist of specialized lipid rafts. The disruption of PD lipid rafts using a sterol metabolism inhibitor reduced PD permeability, potentially by changing the nanostructure of PDs, which is important for the recruitment of PD proteins. In particular, GPI-anchored PD proteins, such as PDBG and PDCB, showed reduced PD localization after the disruption of sterol or sphingolipid metabolism [27] (unpublished data).

The specific location of the PD membrane between cells can make PDs an arena for competition between various apoplasmic signaling factors, such as receptor-like proteins (RLPs) and receptor-like kinases (RLKs) [29]. Many PD RLKs and RLPs were identified in Arabidopsis and rice [30,31]. Some of these proteins also regulate PD permeability. Overexpression of LYSIN MOTIF DOMAIN-CONTAINING GPI-ANCHORED PROTEIN (LYM2), FLAGELLIN SENSING 2 (FLS2), and PD LOCATED PROTEIN (PDLP) family (PDLP5, PDLP1) reduced PD permeability by the accumulation of callose [32,33,34]. STRUBBELIG (SUB)/SCRAMBLED (SCM) is also a PD-localized RLK that interacts with QUIRKY (QKY) at PDs to non-cell-autonomously regulate the morphogenesis of various organs [35]. This system did not change PD permeability, but it was suggested to mediate PD trafficking of unknown mobile signals. Interestingly, PD localized FT-INTERACTING PROTEIN 1 (FTIP1), which is a QKY homolog, functions in FT phloem loading [36].

## 3. PD Connection between Parasitic Plant and Host Plant

Heterografting provides wonderful systems for studying non-cell-autonomous long-distance RNA and protein movement. Notaguchi and his colleagues identified long-distance mobile mRNAs that move from Arabidopsis to *Nicotiana benthamiana* [17]. Natural heterografting can be found in parasitic plant-host interactions. In this special issue, Ekawa and Aoki [37] use a root parasitic plant *Phelipanche aegyptiaca* to investigate inter-phloem connection between host and parasitic plants. Host phloem-expressed GFP can move to *P. aegyptiaca* phloem cells, but haustorial phloem-conducting cells are not conventional sieve elements, since they have no callose-rich sieve plates, but retain nuclei. They showed that the PDs with large size exclusion limits can be formed independently of nuclear degradation and callose deposition. Previously, it was reported that heterografting between different species do not form bona fide phloem connections, but plasmodesma connection was critical between heterospecies.

In the end, I hope that this special issue, “Plasmodesmata and Intercellular Movement”, will provide interested readers with a satisfactory overview of current PD research and attract the interest of other scientists in the field.
